# Ocular surface changes in the treatment of rosacea: comparison between low-dose oral isotretinoin and doxycycline

**DOI:** 10.5935/0004-2749.20200016

**Published:** 2020

**Authors:** Fabio Mendonça Xavier Andrade, Fabiola Rosa Picosse, Laura Pires da Cunha, Camila Maia Valente, Fernanda Machado Bezerra, Helio Miot, Edileia Bagatin, Denise de Freitas

**Affiliations:** 1 Department of Ophthalmology and Visual Sciences, Universidade Federal de São Paulo, São Paulo, SP, Brazil; 2 Department of Dermatology, Universidade Federal de São Paulo, São Paulo, SP, Brazil; 3 Department of Dermatology, Universidade Estadual Paulista “Julio de Mesquita Filho”, São Paulo, SP, Brazil

**Keywords:** Rosacea/drug therapy, Doxycycline/therapeutic use, Doxycycline/administration & dosage, Isotretinoin/therapeutic use, Isotretinoin/administration & dosage, Ocular sur face, Blepharitis, Rosácea/tratamento farmacológico, Doxiciclina/ uso terapêutico, Doxiciclina/administração & dosagem, Isotretinoina/uso terapêutico, Isotretinoina/administração & dosagem, Superfície ocular, Blefarite

## Abstract

**Purpose:**

To compare the impact of ocular changes between systemic treatment with doxycycline and low-dose oral isotretinoin in patients with moderate-to-severe papulopustular rosacea.

**Methods:**

Patients were randomized to receive either isotretinoin 0.3-0.4 mg/kg (group A) or doxycycline 100 mg/ day (group B) for 16 weeks. Ocular symptoms were searched and evaluated, including best-corrected visual acuity (BCVA), Schirmer test, breakup time, rose bengal staining score, and meibomian gland dysfunction grading. The patients were retested at the end of treatment.

**Results:**

The present study included 39 patients (30 females and 9 males). Best-corrected visual acuity was > 20/30 in >90% of patients in both groups and did not change after treatment. After treatment, improvement in ocular symptoms and meibomian gland dysfunction was more pronounced in group B (p<0.05); the other parameters did not reach statistical significance.

**Conclusion:**

Doxycycline improved meibomian gland dysfunction, ocular symptoms, and ocular surface in patients with rosacea. Even though some patients experienced worsening meibomian gland dysfunction and symptoms, no subject experienced any serious complications after administration of low-dose isotretinoin.

## Abstract (pt)

**Objetivos:**

Comparar o impacto das alterações oculares entre o tratamento sistêmico de doxiciclina e isotretinoína em baixa dosagem em pacientes com rosácea papulopustulosa moderada a grave.

**Métodos:**

Os pacientes form randomizados para receber isotretinoína 0,3 a 0,4 mg/kg (grupo A) ou doxiciclina 100mg/dia (grupo B) por 16 semanas. Os sintomas oculares foram pesquisados e avaliados, incluindo melhor acuidade visual corrigida, teste de Schirmer, tempo de ruptura do filme lacrimal, coloração de rosa bengala e graduação da disfunção de glândula de Meibomius. Os pacientes foram novamente testados no final do tratamento.

**Resultados:**

O presente estudo incluiu 39 pacientes (30 mulheres e 9 homens). A melhor acuidade visual corrigida foi >20/30 em >90% dos pacientes em ambos os grupos e não se alterou após o tratamento. A melhora dos sintomas oculares e da disfunção de glândula de Meibomius foi mais pronunciada no grupo B (p<0,05) após o tratamento; as demais variáveis não atingiram significância estatística.

**Conclusão:**

A doxiciclina melhorou a disfunção de glândula de Meibomius, os sintomas oculares e a superfície ocular de pa cientes com rosácea. Mesmo que alguns pacientes tenham piorado a disfunção e os sintomas da glândula de Meibomius, nenhum indivíduo apresentou complicações graves após a admi nistração de baixas doses de isotretinoína.

## INTRODUCTION

Rosacea is a chronic inflammatory disease affecting the facial skin and often the eyes, causing eyelid and ocular surface inflammation. Diagnosis is made based on clinical signs, such as flushing, persistent erythema, papules, pustules, and telangiectasia^(1,2)^.

Rosacea has four subtypes: erythematotelangiectatic, papulopustular, phymatous, and ocular rosacea, each with its own clinical features, though overlap of >1 subtype does occur in the same patient^(1,2)^.

The clinical presentation of ocular rosacea primarily involves meibomian gland dysfunction (MGD) and blepharitis, which leads to evaporative dry eye, conjunctival inflammation, and corneal scarring. Symptoms may include itching, burning, foreign body sensation, blurred vision, and photophobia^(3-5)^.

Although more than half of ocular rosacea cases involve >1 subtype, it is frequently underdiagnosed, either because ocular symptoms are subtle or the skin presentation is overlooked during ophthalmologic evaluation^(3,6)^.

Systemic treatment of ocular rosacea is often indicated when symptoms are refractory to local therapies, including eyelid hygiene, warm compresses, artificial tears, and topical antibiotics. Oral antibiotics with anti-inflammatory effects, such as doxycycline, are typically first-line treatment regimens for MGD^(7,8)^.

Isotretinoin is typically used to treat severe cases of rosacea, and its mechanism of action involves suppressing sebaceous gland activity. Its deleterious effects on the meibomian glands are well known to ophthalmologists and dermatologists worldwide, including its potential to worsen dry eye, especially if the patient already has a history of ocular involvement^(9,10)^.

This study attempted to compare the ocular effects of systemic treatment with doxycycline versus low-dose oral isotretinoin in patients with moderate-to-severe papulopustular rosacea.

## METHODS

The study included patients aged 20-75 years who were diagnosed with moderate or severe papulopustular rosacea, had not used oral antibiotics in the previous three months and had no history of previous oral isotretinoin use. Patients were randomized to receive once-daily oral administration of either isotretinoin 0.3-0.4 mg/kg (group A) or doxycycline 100 mg/day (group B) for 16 weeks.

Before initiating treatment, the presence of blepharitis and specific dry eye-related ocular symptoms was assessed by a patient self-report questionnaire (itching, burning, tearing, and foreign body sensation). Next, an ophthalmologic exam was performed, including best-corrected visual acuity (BCVA), Snellen chart, Schirmer I test without anesthesia, breakup time (BUT), rose bengal staining score, van Bijsterveld scale, and grading the presence of MGD via expressibility and secretion quality (0 - no MGD; 1 - mild; 2 - moderate; 3 - severe) according to the 2011 International Workshop on Meibomian Gland Dysfunction^(11)^. After 16 weeks of treatment, patients answered completed a questionnaire about the same ocular symptoms and were asked if their symptoms had improved, worsened, or remained unchanged since starting the medication. Finally, the ophthalmologic exam was repeated using the same protocol.

Qualitative variables were expressed as percentages. Quantitative variables were represented as means and standard deviation or medians and quartiles (p25-p75) if normality was not demonstrated on the Shapiro-Wilk test. Variables with ordinal characteristics related to the longitudinal behavior of change (improved, unchanged, and worsening) were compared between the groups using the Chi-square test for trend. Quantitative data were compared longitudinally for intra- (T0 × T4) and intergroup (A × B) evolution using a generalized linear mixed effects model with probability adjustment according to the base distribution, autoregressive covariance matrix, and robust estimation of structured covariance. Statistical analysis of the data was performed using IBM SPSS Statistics software, Version 25 (IBM Corp., Armonk, NY, USA), and p<0.05 was considered significant.

All patients provided written informed consent, and the study was approved by the University’s research ethics committee.

## RESULTS

Overall, 39 patients were included (30 females and nine males; mean age, 44.2 years [± 7.9]); 19 were randomized to group A and 20 to group B. Individual findings for each eye were evaluated separately (total of 78 eyes). Clinical and demographic variables are shown in table 1.

**Table 1:** Clinical findings and demographics

Variable	Group A (Isotretinoin)	Group B (Doxycycline)	Total	p-value
Subjects	19	20	39	
Sex				
Female	15 (79%)	15(75%)	30 (77%)	>0.99^a^
Male	4(21%)	5 (25%)	9 (23%)	
Age (years)	44.8 ± 8.0	43.7 ± 8.0	44.2 ± 7.9	0.67^b^
Symptoms	9 (47%)	16 (80%)		0.07^a^
BCVA				
>20/30	35 (92%)	37 (93%)		0.49^c^
20/40-20/60	3 (8%)	1 (2%)		
20/70-20/200	0	1 (2%)		
<20/200	0	1 (2%)		
MGD				
Absent	0	10 (25%)		0.30^c^
Mild	18 (47%)	17(43%)		
Moderate	13 (34%)	6(15%)		
Severe	7(18%)	7(18%)		

BCVA= best-corrected visual acuity; MGD= meibomian gland dysfunction.

a = Fisher exact test;

b = Student *t*-test;

c = Chi-square test for trend.

Before treatment, ocular symptoms (mostly itching and occasional foreign body sensation) were mainly reported by patients in group B (p>0.05). After treatment, more patients in group B reported improvement of symptoms, while more patients in group A reported worsening of symptoms (p<0.01). The evolution of symptoms and clinical findings are shown in table 2.

**Table 2:** Evolution of symptoms and clinical findings

Variables	Group A (Isotretinoin)		P^i^	Group B (Doxycycline)		p-value^i^	p (groups)^ii^
	TO	T4		TO	T4		
Symptoms		4(21%)			13 (65%)		<0.01^a^
Improved		10(53%)			6 (30%)		
Unchanged Worsening		5 (26%)			1 (5%)		
BCVA		2 (5%)			1 (3%)		0.98^a^
Improved		35 (92%)			39 (97%)		
Unchanged Worsening		1 (3%)			0		
Schirmer test (mm)	17.4 ± 12.8	17.0 ± 12.8	0.46^b^	18.2 ± 11.7	20.2 ± 12.3	0.57^b^	0.38^b^
RBSS	33 (87%)	34 (89%)	0.79^b^	32 (80%)	36 (90%)	0.45^b^	0.47^b^
0	5 (13%)	4(11%)		8 (20%)	4 (10%)		
>0							
BUT	5.2 ± 4.1	5.6 ± 3.5	0.11^b^	3.6 ± 2.6	5.9 ± 4.3	<0.01^b^	0.76^b^
MGD		14 (37%)			18(45%)		0.03^a^
Improved		15 (39%)			22 (55%)		
Unchanged Worsening		9 (24%)			0		

BCVA= best-corrected visual acuity; RBSS= rose bengal staining score; BUT= breakup time; MGD= meibomian gland dysfunction.

a = Chi-square test for trend;

b = Generalized linear mixed effects model;

i = p (T0 × T4);

ii = p (group A × group B).

In both groups, BCVA was 20/30 or better in >90% of patients and did not differ after treatment.

Before treatment, the mean Schirmer test scores were similar in both groups (17.4 mm ± 12.8 mm in group A and 18.2 mm ± 11.1 mm in group B); however, these values changed after treatment (17.0 mm ± 12.8 mm in group A and 20.2 mm ± 12.3 mm in group B) (p>0.05).

Before treatment, the mean BUT was 5.4 s ± 4.1 s in group A and 3.6 s ± 2.6 s in group B (p<0.05). After treatment, group A exhibited a slight increase (5.6 s ± 2.5 s; p=0.11) and there was a considerable increase in group B (5.9 s ± 4.3 s; p<0.01).

Before treatment, the rose bengal staining score was 0 in 33 (87%) patients in group A and 32 (80%) in group B (p>0.05). After treatment, 34 (89%) patients in group A had a grading score of 0 in addition to 36 (90%) in group B (p>0.05).

The MGD grading classification was not evenly distributed between groups since it was randomized correctly. Only group B had cases with grade 0 MGD, while group A had more cases with grade 2 MGD. Nevertheless, after treatment, group B showed more improvement in MGD, and group A had more cases of worsening symptoms (p<0.05). We performed a separate analysis of only those cases with MGD, and the statistics still indicated the same results (p<0.05).

## DISCUSSION

Ocular rosacea is a serious diagnosis and should be considered when examining a patient with MGD. Ophthalmologists must realize that ocular findings can precede skin involvement, and dermatologists must consider that the severity of ocular manifestations is not related to the severity of the skin disease. Rosacea patients are at risk for severe ocular surface alterations and corneal impairment, including possible blindness; therefore, specialists are sometimes reluctant to administer medications, such as isotretinoin, that can reduce meibomian gland function and consequently damage the ocular surface^(1,5)^.

The ocular side effects of isotretinoin include conjunctivitis, hordeolum, blepharitis, keratitis, lower Schirmer test scores, and others. However, it is also one of the best treatment options for severe rosacea. Low-dose reg imens (<0.5 mg/kg) have been studied and indicate milder side effects^(9,12-14)^.

Doxycycline is a second-generation tetracycline known to improve meibomian gland production and increase tear film stability; various studies report good results in rosacea patients. Gastrointestinal upset is a common adverse effect, occurring in 0.54%-51.7% of such patients^(15)^.

In our study, most ocular symptoms before treatment were reported by patients in group B, which also had more cases of grade 0 MGD. This is probably because ocular symptoms are not always directly related to the severity of eyelid alterations. In spite of this, group B demonstrated a higher rate of improvement, and no cases experienced worsening symptoms (p<0.01), emphasizing the benefits of doxycycline on MGD.

Other studies have reported a transient reduction in Schirmer test scores and BUT with isotretinoin use. Patients in group A had a minor decrease (0.4 mm) in the Schirmer test scores, but the mean value after treatment was still above dry eye levels (p>0.05). However, the mean value of BUT improved slightly after treatment in group A and considerably in group B (p<0.01)^(14,16)^.

The rose bengal staining score did not change considerably in either group after treatment since most pa tients scored 0 (no ocular surface alteration) before and after treatment (p>0.05). This is very important, as keratitis is the most sight-threatening side effect of ocular rosacea^(3)^.

After treatment, group B exhibited more improvement in MGD compared to group A. In addition, the only cases of worsening MGD occurred in group A. Some studies have indicated doxycycline’s restorative properties on meibomian gland secretion, even at a molecular level, restoring the lipid components and improving MGD^(7,15,17)^.

Even though doxycycline is more effective in treating the ocular manifestations of rosacea, no serious ocular complications were seen after low-dose isotretinoin treat ment. Rose bengal staining scores, Schirmer tests, and BUT did not reach concerning levels, and BCVA remained unchanged in both groups. It must be considered that none of the patients in the present study had any corneal impairment before treatment, as such cases are more likely to be susceptible to ocular surface changes.
